# Congenital Immature Grade ΙΙΙ Teratoma of the Neck: A Case Report

**DOI:** 10.7759/cureus.44373

**Published:** 2023-08-30

**Authors:** Nazneen Liaqat, Israr Ud Din, Zeeshan Ali, Majid Rashid, Afsheen Liaqat

**Affiliations:** 1 Department of Otorhinolaryngology, Head and Neck Surgery, Khyber Teaching Hospital (KTH), Peshawar, PAK; 2 Department of Medicine, Khyber Medical University, Peshawar, PAK; 3 Department of Internal Medicine, Khyber Teaching Hospital (KTH), Peshawar, PAK; 4 Department of Hematology, Armed Forces Bone Marrow Transplant Center, Rawalpindi, PAK

**Keywords:** congenital disease, cervical tumor, pediatric head and neck surgery, head and neck tumors, immature teratoma

## Abstract

Teratoma is a rare type of germ cell tumor that consists of structures derived from all three germ layers of the embryo with varying proportions. While most of these are benign, some can turn malignant. The most common location of teratomas is the sacrococcygeal region, while their occurrence in the neck region is very rare. Broadly classified, immature teratomas contain poorly differentiated tissues, while mature ones have well-differentiated tissues. Here, the authors present a case of a 12-month-old child who presented with a huge neck mass. Radiological imaging studies were performed. Under a multidisciplinary team approach, the child was treated successfully with surgical excision. Histopathology revealed the mass to be an immature teratoma of grade III. Postoperatively, no recurrence has been noted.

## Introduction

Teratoma, by definition, is a type of congenital germ cell tumor containing a variety of tissues, such as hair, muscle, and bone, derived from all three germ layers [1]. These tumors are typically found in the midline regions at intra or extragonadal locations [1]. Overall prevalence of teratomas is rare constituting almost 1/13000 births worldwide [2]. They are usually located in the sacrococcygeal area whereas cervical teratomas constitute only two to nine percent of congenital teratomas showing their extremely rare occurrence [3].

On the basis of the degree of differentiation of their components, teratomas are classified into mature and immature. Mature teratomas contain well-differentiated tissues while immature teratomas contain immature, non-malignant tissues [4]. Mature teratomas are more frequent in occurrence than immature ones [4]. Although rare, congenital cervical teratomas can pose dire consequences including perinatal asphyxia, dysphagia, feeding difficulties, respiratory distress, cosmetic disfigurement, etc. via their mass effect on aerodigestive tract [5]. The authors, here, report a case of congenital immature grade III teratoma of the neck region that was successfully managed with complete surgical excision.

## Case presentation

**Figure 1:**
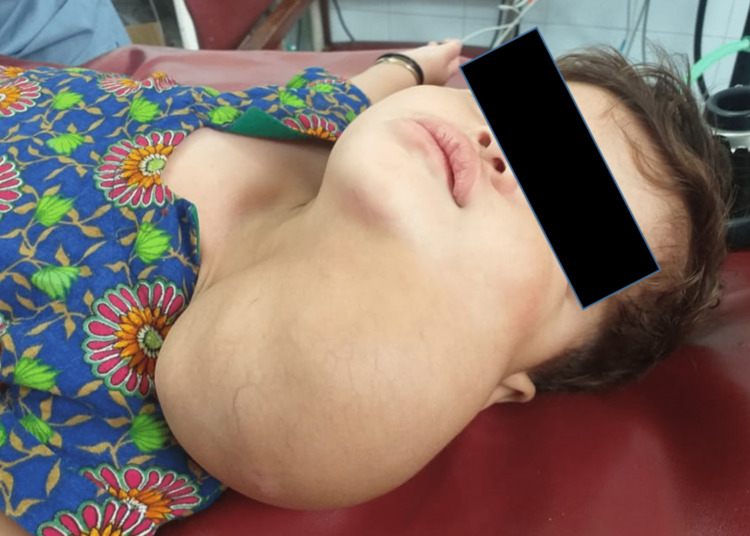
Preoperative picture of the child with left lateral neck mass.

A 12-month-old girl presented on an outpatient basis with the complaint of a huge neck mass since birth. According to the guardian of the child, she manifested this growth since her birth which, with time, gradually increased in size and restricted the mobility of her neck. The general condition of the patient was satisfactory and she was active. On physical examinations, the mass was firm, 10×8×6 cm in size, extending from the left submandibular region, covering the entire left aspect of the neck, and pushing the ipsilateral mandible to the opposite side (Figure 1). The overlying skin was intact. Due to the mass effect, the neck was tilted to the opposite side with restricted mobility. On examinations, no neurological deficits were noted.

Ultrasonography of the neck showed a huge cystic mass with internal small solid components located in the left anterolateral aspect of the neck extending the entire length of the neck. It was approximately 7.6×6.1×9.2 cm in size, with 223 mL volume, having an exophytic extension. Color Doppler ultrasound noted vascularity at certain places in solid components.

**Figure 2:**
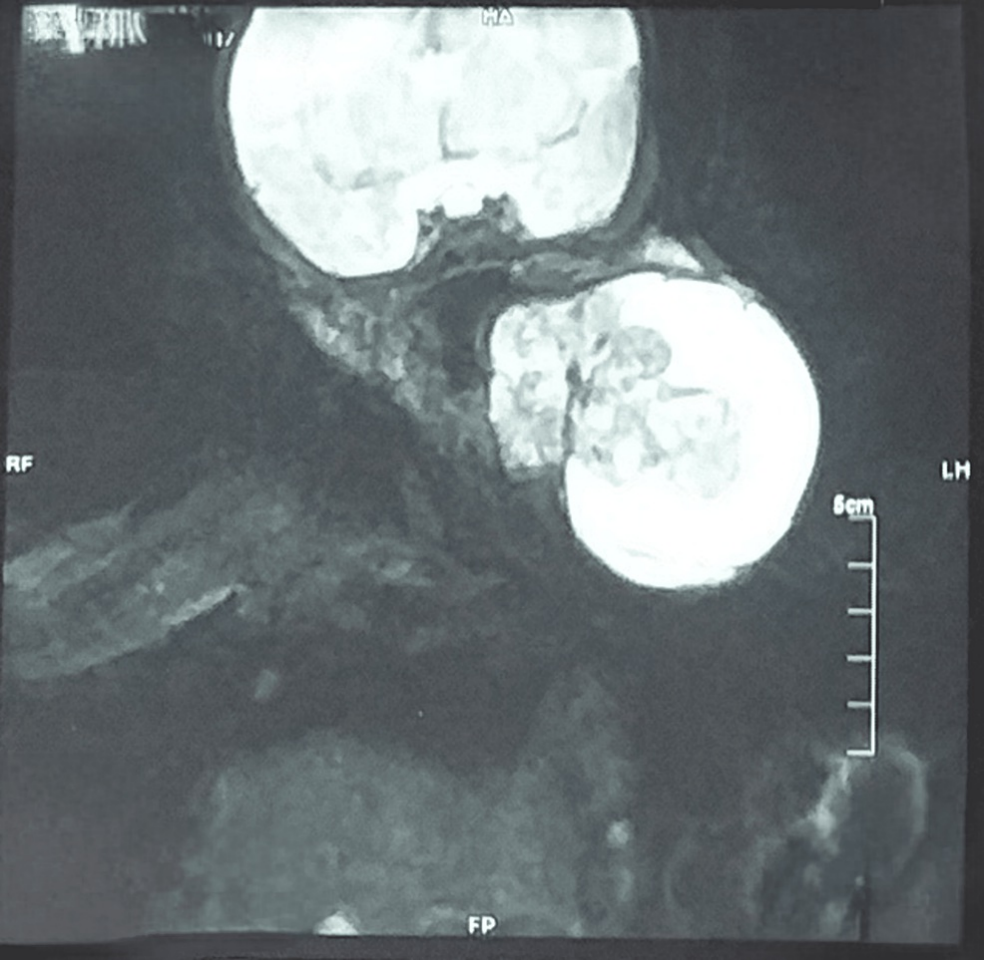
T1 weighted MRI coronal view after contrast enhancement showing complex solid and cystic lesion in left submandibular region, extending over left lateral aspect of neck.

Multiplanar imaging with contrast enhancement was done via MRI of the brain and neck acquiring T1/T2 weighted and proton density sequences. A complex solid and cystic lesion was noted in the MRI, originating in the left submandibular region and extending over the left lateral aspect of the neck (Figure 2). It was compressing and displacing the oropharynx. The lesion could not be seen separately from the left lobe of thyroid.

Detailed case discussion was done and surgical excision was proposed by the multidisciplinary team. Under general anesthesia, the mass was excised completely without any disruption of the walls through a transcervical approach. Preoperatively, the mass was found to be well-circumscribed with irregular contours and had no adhesions with any of the surrounding structures, including the thyroid.

**Figure 3:**
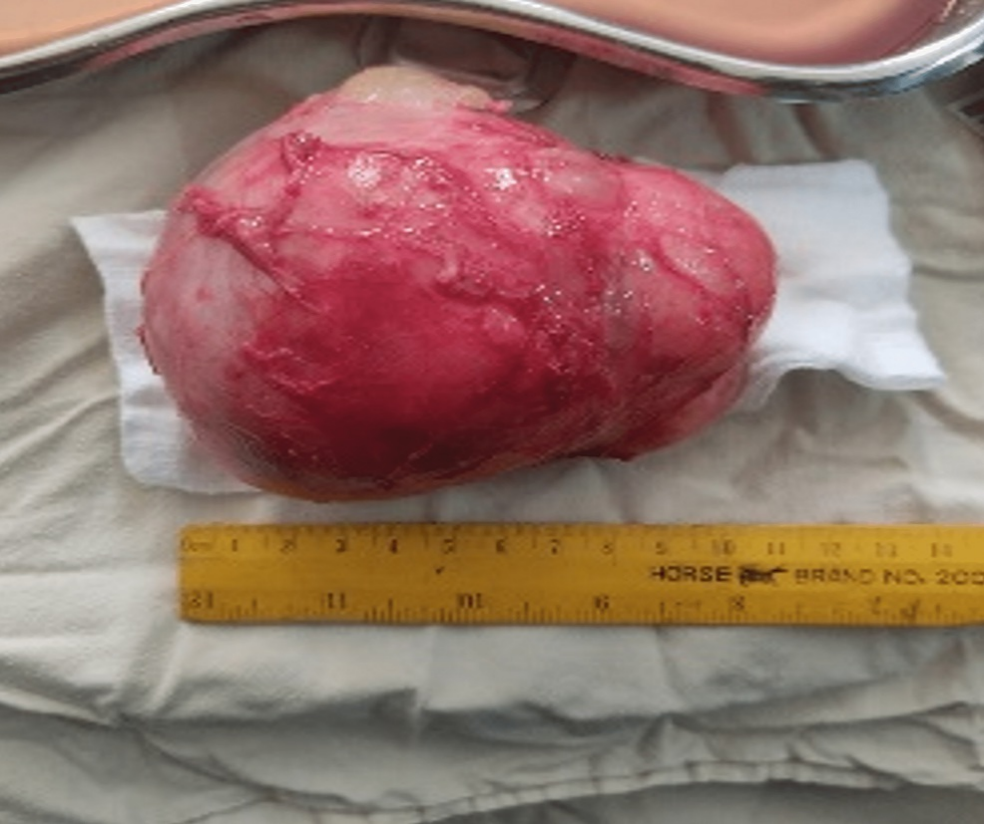
Completely excised mass.

Grossly, the lump was 9.2×8.8×5.3 cm, weighing 418 g. It was well-circumscribed, lobulated, and gray-white (Figure 3). Cut sections revealed both solid and cystic components with a focal area of cheesy material with hair. Microscopy showed a neoplasm composed of variable amounts of mature elements from all three germ layers admixed with immature neuroectodermal elements. The cells appeared primitive with scant cytoplasm, hyperchromatic nuclei, and frequent mitoses. Histopathological impression of immature teratoma of grade III was made.

This case is limited by the unavailability of actual ultrasound images and histological slides. While corresponding reports exist, the lack of visual representation hinders a comprehensive analysis. The findings primarily rely on textual descriptions, MRI, and clinical information. However, for the sake of readers' comprehension, authors may present histological data from another study in the case presentation section to enhance understanding. The patient has been followed for nine months postoperatively, without a recurrence.

## Discussion

The exact etiology of teratoma is not known although their multifactorial etiology is a complex of the chromosomal abnormalities, gene mutations, and the irregularities of embryonic development [6]. Traditionally, teratomas have been considered almost always benign but the newer literature suggests otherwise, that is their malignant potential is also appreciated, with less than five percent of cervical teratomas being malignant in nature [6].

**Figure 4:**
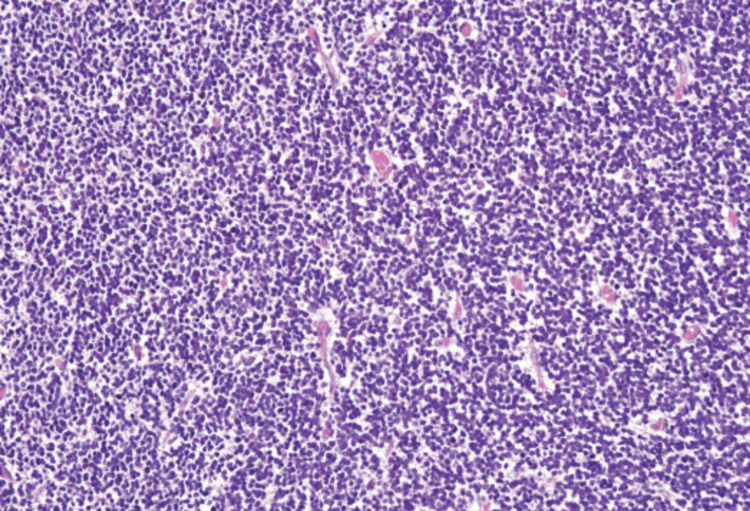
Immature grade III teratoma. Grade III cellularity refers to a level of cell density similar to that found in the germinal matrix of the fetal brain or the granular layer of the normal adult cerebellum. In this grade, nuclei are in close proximity, touching each other, or with an inter-nuclear distance shorter than 1 nuclear diameter [8].

Histologically, they are classified as mature and immature. Mature teratomas usually contain well-differentiated tissues from the three germ cell layers: ectoderm, mesoderm, and endoderm. Immature teratomas also contain tissues from all the germ cell layers, but immature/fetal tissues, primarily neuroepithelial tissues, are present (Figure 4) [7]. Mature teratomas are found more frequently than their immature counterparts [4]. Although immaturity does not equate to malignancy; however, immature teratoma is associated with larger tumor size, higher alpha-fetoprotein levels, and increased requirement for airway management as compared to mature teratoma [5]. Another study has suggested a higher risk of relapse with immature teratoma lesions as compared to mature ones [4].

Despite being mostly benign, teratomas of the head and neck region can cause life-threatening complications, both antenatal and postnatal. Main antenatal complications are polyhydramnios, airway compression, risk of tumor rupture, delayed normal growth of the fetus, hypotrophy, and prematurity. Antenatal diagnosis using ultrasonography during 15-16 weeks of pregnancy can be helpful in deciding the mode of delivery [9]. There was no antenatal record available in this reported case.

The differential diagnosis of teratomas of the head and neck includes hemangiomas, cystic hygromas, lymphangiomas, lipomas, dermal cysts, vascular malformations, and cutaneous cysts. Regarding diagnostic radiology, ultrasound can be used as a first-line investigation to determine the nature, consistency, and size of the lump. While MRI is considered as best imaging modality [1]. Radiological features supportive of teratoma include cystic and solid components, heterogeneous appearance, presence of calcification, absence of ipsilateral thyroid lobe, or splaying of the thyroid gland near the mass [1,6]. Calcifications of the mass detected on preoperative CT scan can also give a clue of the diagnosis of teratoma [10].

Early intervention under a multidisciplinary approach, complete surgical excision (both macroscopically and microscopically) with close and meticulous follow-up is considered the optimal treatment for all cervical teratomas [4]. Follow-up should include clinical assessment and MRI. Immature teratoma and incomplete excision are considered risk factors for recurrence [5].

## Conclusions

Congenital cervical teratoma, a rare and benign condition, can have severe consequences for infants. However, a collaborative multidisciplinary approach, involving airway management and early single-staged surgical excision, offers complete treatment and a high survival rate. The combined efforts of healthcare specialists ensure a thorough evaluation of the tumor, considering size, location, and potential complications. Prompt airway management is essential, and early surgical intervention provides a definitive solution, improving long-term outcomes for patients.

Congenital cervical teratoma requires a coordinated team effort to achieve the best results. Its prognosis mostly depends on the risk of neonatal respiratory distress, its extension, and potential malignancy. Surgical management must be as complete as possible to avoid recurrences and malignant transformation. By addressing airway concerns and performing an early single-staged surgical excision, healthcare professionals can effectively treat the condition and increase the chances of a successful recovery. This comprehensive approach, involving various specialists, ensures a thorough evaluation and provides infants diagnosed with congenital cervical teratoma with the best possible care, leading to favorable outcomes and a promising survival rate.
